# Circular Tessera Codes in the Evolution of the Genetic Code

**DOI:** 10.1007/s11538-020-00724-z

**Published:** 2020-04-04

**Authors:** Elena Fimmel, Martin Starman, Lutz Strüngmann

**Affiliations:** Institute of Mathematical Biology, Faculty for Computer Sciences, Mannheim University of Applied Sciences, 68163 Mannheim, Germany

**Keywords:** Genetic code, Degeneracy, Circular code, Tessera

## Abstract

The origin of the modern genetic code and the mechanisms that have contributed to its present form raise many questions. The main goal of this work is to test two hypotheses concerning the development of the genetic code for their compatibility and complementarity and see if they could benefit from each other. On the one hand, Gonzalez, Giannerini and Rosa developed a theory, based on four-based codons, which they called tesserae. This theory can explain the degeneracy of the modern vertebrate mitochondrial code. On the other hand, in the 1990s, so-called circular codes were discovered in nature, which seem to ensure the maintenance of a correct reading-frame during the translation process. It turns out that the two concepts not only do not contradict each other, but on the contrary complement and enrichen each other.

## Introduction

In 1986, John Maynard Smith stated: “We understand biological phenomena only when we have invented machines with similar properties” (Smith 1986, pp 99–100). This quotation explains the motivation of this work quite well. This paper was written in order to better understand the origin of the genetic code using such a machinery. One possible machine or rather a model which gives a feasible explanation for an important aspect of the evolutionary processes of the genetic code was found by Gonzalez, Giannerini and Rosa. In their work “On the origin of degeneration in the genetic code” (Gonzalez et al. 2019) they focus on the degeneracy of amino acid coding and especially on symmetry as an essential cause and consequence of the natural phenomena of degeneracy (compare also Fimmel and Strüngmann 2016). A famous example, which shows the importance of including symmetry deliberations when considering natural phenomena, can be found in quantum mechanics. Here, symmetry describes more than just the patterns that matter takes – it is used to classify the nature of quantum states. This is by far not the only example of its kind. Noether’s theorem even states a one-to-one connection between fundamental laws of nature - so-called conservation laws- and respective symmetries in nature.

Taking these general considerations into account, Gonzalez, Giannerini, and Rosa argue that none of the theories regarding the origin of the genetic code pays the necessary attention to the idea of symmetry (Gonzalez et al. 2019). As a consequence the concept of tessera codes was developed. The tesserae build a subset of all tetranucleotides, chosen in such a way that the degeneracy of the vertebrate mitochondrial genetic code can be explained from the symmetries of the tesserae (Gonzalez et al. 2012).

The other line of thought adressed by the current work is the theory of circular codes. This theory is intended to explain the property of the noise-immunity of the genetic code, and is based on a proposal by Crick et al. (1957). They argue that the coding of amino acids requires only a subset of codons where the correct reading-frame is automatically and immediately recognizable - the so-called comma-free property. While Crick’s theory was refuted in reality (Nirenberg and Matthaei 1961), 40 years later so-called circular codes were discovered in nature (Arqués and Michel 1996). More specifically, it has been noticed that the set of codons, which, together with their frame-shifts in three potential reading-frames, are the most commonly used across all species, has very remarkable properties in terms of detecting the correct reading-frame (Fimmel and Strüngmann 2018; Fimmel et al. 2016; Michel 2017). The comma-free codes proposed by Crick belong to the same family of circular codes, but within them they have the most distinct error-detecting properties (see, for instance, Fimmel et al. 2018, 2017, 2016, xxxx). The natural circular codes have even more interesting structural properties, which makes it very doubtful that these structures play no role in biological processes (Arqués and Michel 1996, Fimmel and Strüngmann (2018)).

The primary goal of this work is to combine the two concepts, tesserae and circular codes, and see if they could benefit from each other. In this work we specify among other things a construction algorithm for circular tessera codes of maximal length. Furthermore, self-complementary tessera codes are characterized and criteria for their self-complementarity are formulated and proved in the language of graph theory. The growth tables for circular and comma-free tessera codes are also presented for the first time. In summary, one result of the work is that the two concepts under scrutiny—that of tessera codes and circularity—have proved to be mutually compatible and complementary.

Thus, with this work we hope to bring more clarity into the possible role of tesserae in the evolutionary process of the genetic code and the mechanisms behind it.

## Definitions and Notations

The genetic code is written with words of three letters called *codons*, built over an alphabet

$$\begin{aligned} \mathcal {B}:=\{U(T), C, A, G\} \end{aligned}$$

of four letters which are called *nucleotide bases* Uracil (Thymine), Cytosine, Adenine, and Guanine, in short *U*(*T*), *C*, *A*, *G*. Clearly, the number of codons is $4^3=64$ and by $|\mathcal {B}^3|$ we will denote the cardinality of the set $\mathcal {B}^3$. Accordingly, the set $\mathcal {B}^2$ denotes the set of 16 *dinucleotides* and the set $\mathcal {B}^4$ contains the 256 *tetranucleotides*. It is hypothesized that during evolution the genetic code had several ancestors that might have consisted not only of trinucleotides but of dinucleotides or tetranucleotides or even combinations of these (see Baranov et al. 2009; Gonzalez et al. 2012; Seligmann 2014; Patel 2005; Wilhelm and Nikolajewa 2004; Wu et al. 2005). In particular, in Gonzalez et al. (2012) the *tessera code* was suggested as an ancestral code that might have been the origin of the mitochondrial code (see also Gonzalez et al. 2019). In order to define the tessera code we have to introduce some group theory and how it can be applied in the genetic setting.

### Klein Four-Group and Equivalence Classes of Dinucleotides

The *symmetric group* on a set of elements is usually known as the group of *permutations* of these elements. Applying this to our genetic alphabet $\mathcal {B}$ we define the symmetric group $S_{\mathcal {B}}$ as

$$\begin{aligned} S_{\mathcal {B}}= \{ \pi : \mathcal {B}\rightarrow \mathcal {B}\mid \pi {\,\,is\,\,\,bijective} \} \end{aligned}$$

with the usual group operation given by composition of functions. Recall that a *group* $(H, \circ )$ is a set *H* together with an operation $\circ : H \rightarrow H$ such that $\circ$ is associative and *H* contains a neutral element *e* as well as inverses $h^{-1}$ for all $h \in H$ (see Rotman 1995 for more details on groups). The group $S_\mathcal {B}$ has $4!=24$ elements and is trivially isomorphic to the symmetric group $S_4$ on four elements. We will use standard notation as can be found in Rotman (1995), e.g. we will either write $\pi =(A,G,C,U)$ or $\pi : (A,U,C,G) \rightarrow (G,A,U,C)$ if $\pi$ satisfies $\pi (A)=G, \pi (U)=A, \pi (C)=U$, and $\pi (G)=C$. Naturally, any permutation $\pi :\mathcal {B}\rightarrow \mathcal {B}$ can be applied to *n*-nucleotides of any length componentwise, i.e. if $x=b_1 \cdots b_n \in \mathcal {B}^n$, then $\pi (x)=\pi (b_1) \cdots \pi (b_n)$. There is no danger of confusion when denoting the induced bijective map $\mathcal {B}^n\rightarrow \mathcal {B}^n$ by $\pi$ again for any natural number *n*.

In Fimmel et al. (2014), Fimmel et al. (2015) a subgroup *L* of $S_{\mathcal {B}}$ was identified that seems to play an important role in error-detection and error-correction mechanisms during the translation process. This group consists of all permutations from $S_{\mathcal {B}}$ that preserve the codon-anticodon relation and can be geometrically interpreted as the symmetry group of a square. In particular, it contains 4 bijective transformations of nucleotide bases that are invariant with respect to the chemical characters of the nucleotides (we will use the notations from Fimmel et al. 2014, 2015). These are the

Identity:

$$\begin{aligned} \text {I (or } id ) :(A, U, C, G) \rightarrow (A, U, C, G); \end{aligned}$$

Strong/Weak (SW) or complementary transformation:

$$\begin{aligned} \text {SW (or } \textit{c}) :(A, U, C, G) \rightarrow (U, A, G, C); \end{aligned}$$

Pyrimidine/Purine (YR) transformation:

$$\begin{aligned} \text {YR (or } \textit{p}) :(A, U, C, G) \rightarrow (G, C, U, A); \end{aligned}$$

and Keto/Amino (KM) transformation:

$$\begin{aligned} \text {KM (or } \textit{r}) :(A, U, C, G) \rightarrow (C, G, A, U). \end{aligned}$$

**Fig. 1:**
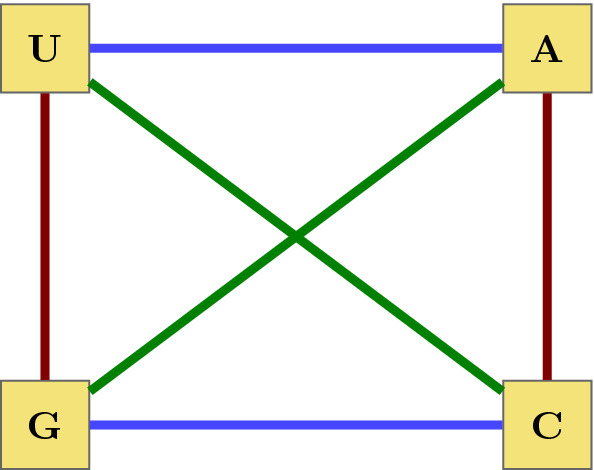
Graphical representation of the primeval base symmetries. *KM* is represented by red, *YR* by green and *SW* by blue colored lines (Color figure online)

In particular, the *complementary map* *c* is biologically important since it mirrors the hydrogen bonds $A \leftrightarrow T$ and $C \leftrightarrow G$ of the DNA double helix. Moreover, the transformation *r* from above carries codons of degeneracy class 4 to codons of degeneracy class less than 4 and vice versa - a symmetry property of the genetic code that was already observed by Rumer (see Fimmel et al. 2014, 2015 for more details). In the sequel we will denote the set of these four transformations as $\mathcal {V}=\{I, SW, YR, KM\}$ (Fig. 1).

Equipped with the usual group operation of $S_{\mathcal {B}}$ the set $\mathcal {V}$ forms a subgroup of the symmetric group $S_{\mathcal {B}}$ which is isomorph to the so-called *Klein four-group*. It can be easily verified that the group $\mathcal {V}$ is commutative, i.e. $\alpha \circ \beta = \beta \circ \alpha$ for all $\alpha , \beta \in \mathcal {V}$ and that all permutations in $\mathcal {V}$ are of order two, i.e applying them twice yields the identity $\alpha \circ \alpha =id$ for every $\alpha \in \mathcal {V}$.

As we will see in the next section, the group $\mathcal {V}$ is used in order to define the class of tesserae in mathematical terms. If we consider $\mathcal {V}$ acting on the set of dinucleotides $\mathcal {B}^2$ we obtain four *orbits* of size four. Recall that an orbit of an element *x* (here a dinculeotide) under some group *H* (here $\mathcal {V}$) is defined as $[x]=\{h(x) : h \in H \}$. Each orbit represents an equivalence class under the natural equivalence relation $d_1d_2 \sim d_1'd_2'$ if and only if there is $\pi \in \mathcal {V}$ such that $\pi (d_1d_2)=d_1'd_2'$. An easy observation shows that for each such equivalence class there is a unique transformation $\pi \in \mathcal {V}$ that maps the first nucleotide of a dinucleotide in that class to the second nucleotide, e.g. the map *SW* for the class $[AU]=\{AU,UA,CG,GC \}$. Table 1 below shows the four equivalence classes and the corresponding permutations.

**Table 1:** Each column is one of the four equivalence classes of dinucleotides: $\Sigma _I=[AA]$, $\Sigma _{SW}=[AU]$, $\Sigma _{YR}=[AC]$, $\Sigma _{KM}=[AG]$ under the action of $\mathcal {V}$ on $\mathcal {B}^2$

$\mathcal {V}$	$\Sigma _{I}$	$\Sigma _{SW}$	$\Sigma _{YR}$	$\Sigma _{KM}$
*I*	AA	AU	AC	AG
*SW*	UU	UA	UG	UC
*YR*	CC	CG	CA	CU
*KM*	GG	GC	GU	GA

The left most column shows the transformation that sends the first dinucleotide in the class to the second, third and fourth, respectively, e.g. $YR(AA)=CC$. The column header are the equivalence classes names. The header index is the unique transformation used for mapping the first nucleotide of a dinucleotide to the second

We are now almost in the position to define the set of tesserae as introduced in Gonzalez et al. (2012). But before we need some more technicalities. Besides the group $S_{\mathcal {B}}$ acting as a group of exchanges of bases, there is a second important group which consists of transformations that permute the positions of single bases in a nucleotide sequence. Together with the usual composition $\circ$ of maps these permutations form again a group that once more can be seen as a symmetric group $S_n$. For the convenience of the reader we here only recall the biologically relevant permutations that will be of importance for us: the so-called *reversing permutation* and the $n-1$ *shift operations* $\alpha _1, \cdots , \alpha _{n-1}$. Given an *n*-nucleotide $x=N_1 \cdots N_n$ we define $\overleftarrow{{{^{}}}}$ and $\alpha _k$ for $k \le n-1$ as

$$\begin{aligned} \overleftarrow{{N_1 \cdots N_n}}=N_n \cdots N_1,\quad \alpha _k(x)=N_{k+1} \cdots N_nN_1 \cdots N_k \end{aligned}$$

which are the *n*-nucleotides obtained from *x* by reversing or a shift of *k* positions, respectively. Explicitely, for $n=4$ we have

$$\begin{aligned} \overleftarrow{{{N_1N_2N_3N_4}}}=N_4N_3N_2N_1 \end{aligned}$$

and

$$\begin{aligned}&\alpha _1(N_1N_2N_3N_4)=N_2N_3N_4N_1 \quad \\&\quad \alpha _2(N_1N_2N_3N_4)=N_3N_4N_1N_2 \quad \alpha _3(N_1N_2N_3N_4)=N_4N_1N_2N_3 \end{aligned}$$

It is now obvious that the anti-n-nucleotide of some n-nucleotide *x* can be described as $\overleftarrow{{SW(x)}}$ with the complementary map *SW* from $\mathcal {V}$. For trinucleotides (codons) it is well-known that the anti-codon is always different from the codon. However, if *n* is even it might happen that $\overleftarrow{{SW(x)}}=x$ for some *n*-nucleotide *x*. These nucleotide sequences are called *self-complementary*. For example, if $n=4$, then the tetranucleotide *ACGU* is self-complementary since $\overleftarrow{{SW(ACGU)}}=\overleftarrow{{UGCA}}=ACGU$.

### Tesserae: Definition and Structure

Tesserae were motivated biologically in an evolutionary context in Gonzalez et al. (2012). Each tessera is a tetranucleotide that has a particular form that comes from the symmetries induced by the group $\mathcal {V}$. Let us give a definition of a tessera in mathematical terms (see also Gonzalez et al. 2012 and Fimmel and Strüngmann 2019):

#### Definition 2.1

A *tessera* is a tetranucleotide (four letter word) $t \in \mathcal {B}^4$ of the form

$$\begin{aligned} t = N_1N_2 \alpha (N_1) \alpha (N_2) \end{aligned}$$

where $N_1, N_2 \in \mathcal {B}$ and $\alpha \in \mathcal {V}$. The set of all valid tesserae is denoted by *TESS*.

The set *TESS* is also called the *tessera code* since it is a subset of $\mathcal {B}^4$ and hence a code in the sense that every concatenation of words from *TESS* has a unique decomposition over *TESS*. Clearly, the size of *TESS* is 64 and so we have $|TESS|=|\mathcal {B}^3 |$. Table 2 shows the set of all tesserae together with their generating transformation.

**Table 2:** The table of all tessera with the generating transformation

Dinucleotide	*id*	*c*	*p*	*r*
*AA*	*AAAA*	*AAUU*	*AAGG*	*AACC*
*CC*	*CCCC*	*CCGG*	*CCUU*	*CCAA*
*GG*	*GGGG*	*GGCC*	*GGAA*	*GGUU*
*UU*	*UUUU*	*UUAA*	*UUCC*	*UUGG*
*AC*	*ACAC*	*ACUG*	*ACGU*	*ACCA*
*AG*	*AGAG*	*AGUC*	*AGGA*	*AGCU*
*AU*	*AUAU*	*AUUA*	*AUGC*	*AUCG*
*CA*	*CACA*	*CAGU*	*CAUG*	*CAAC*
*CG*	*CGCG*	*CGGC*	*CGUA*	*CGAU*
*CU*	*CUCU*	*CUGA*	*CUUC*	*CUAG*
*GA*	*GAGA*	*GACU*	*GAAG*	*GAUC*
*GC*	*GCGC*	*GCCG*	*GCAU*	*GCUA*
*GU*	*GUGU*	*GUCA*	*GUAC*	*GUUG*
*UA*	*UAUA*	*UAAU*	*UACG*	*UAGC*
*UC*	*UCUC*	*UCAG*	*UCCA*	*UCGA*
*UG*	*UGUG*	*UGAC*	*UGCA*	*UGGU*

It is easy to see that a codon $N_1N_2N_3 \in \mathcal {B}^3$ can be uniquely extended to a valid tessera $tess(N_1N_2N_3)=N_1N_2N_3N_4$ by determing the unique permutation $\alpha \in \mathcal {V}$ such that $\alpha (N_1)=N_3$ and letting $N_4=\alpha (N_3)$. This shows that the tessera code *TESS* is 1-error-correcting and it was shown in Fimmel and Strüngmann (2019) that *TESS* can be obtained as a linear code from $\mathcal {B}^3$ and by the so-called *Plotkin construction* from $\mathcal {B}^2$ - for more details on this see (Fimmel and Strüngmann 2019).

**Fig. 2:**
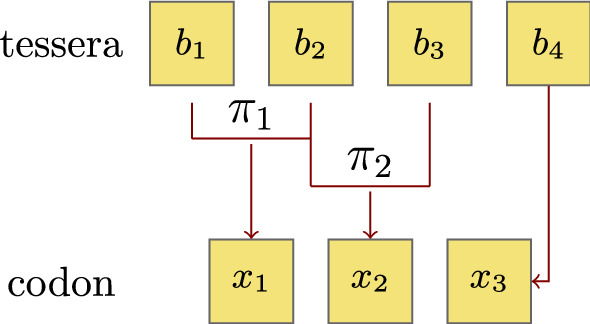
Schematic representation of the mapping between the tessera $b_1$ $b_2$ $b_3$ $b_4$ onto the codon $x_1$ $x_2$ $x_3$. (Color figure online)

In Gonzalez et al. (2012) the idea of symmetric primeval adaptor molecules that could recognize the normal reading frame in the coding strand in the 3$\prime$–5$\prime$ direction, in the complementary strand in the 3$\prime$–5$\prime$ direction, in the coding strand in the reverse 5$\prime$–3$\prime$ direction and in the complementary strand in the reverse 5$\prime$–3$\prime$ direction was utilized to propose an ancient model of tRNA adaptors that explains the reading mechanism and degeneracy distribution of the tesserae. In particular, since there exist self-complementary tesserae, e.g. *ACGU*, the tessera code allows degeneracy 2 and 4 only. Maintaining the degeneracy an algorithm was suggested in Gonzalez et al. (2019) for passing from the tessera code back to the (mitochondrial) genetic code in the following way: We assign to each of the transformations from $\mathcal {V}$ a letter in the genetic alphabet via $I \leftrightarrow A$, $SW \leftrightarrow U$, $KM \leftrightarrow C$ and $YR \leftrightarrow G$ and then perform the following algorithm displayed in Fig. 2.

For instance, the tessera *ACGU* will be mapped to the codon *CUU* since $KM(A)=C$ and $SW(C)=G$. In the sequel we will denote by $cod(N_1N_2N_3N_4)$ the corresponding codon under this algorithm. However, note that the two mappings $tess(\cdot )$ and $cod(\cdot )$ are not inverses of each other.

We now aim for a better description of tesserae. Let us assume that $N_1N_2N_3N_4$ is a tessera. By definition there is an element $\alpha \in \mathcal {V}$ such that

$$\begin{aligned} N_3N_4=\alpha (N_1N_2). \end{aligned}$$

This implies that $N_1N_2$ and $N_3N_4$ have to be in the same equivalence class $\Sigma _{\alpha }$ displayed in Table 1. Thus, the tessera code can be split into four disjoint subsets.

$$\begin{aligned} TESS = TESS_{I} \cup TESS_{SW} \cup TESS_{YR} \cup TESS_{KM} \end{aligned}$$

where

$$\begin{aligned} TESS_i = \{d_1d_2\in TESS \mid d_1,d_2\in \Sigma _{i}\} \textit{ for }i\in \{I,SW,YR,KM\}. \end{aligned}$$

Clearly, any subset $X \subseteq TESS$ has a similar induced decomposition where the components could be empty.

#### Definition 2.2

Let $X\subseteq \mathcal {B}^4$ be a tessera code. Then

$$\begin{aligned} X = X_{I} \cup X_{SW} \cup X_{YR} \cup X_{KM} \end{aligned}$$

where

$$\begin{aligned} X_i = X \cap TESS_i \textit{ for }i\in \{I,SW,YR,KM\}. \end{aligned}$$

The above decomposition will be used in Sect. 4 for constructing all maximal circular tessera codes.

### Graph Theoretical Approach

In this section we recall a graph theory approach from Fimmel et al. (2016) that turned out to be very useful for determining properties of circular codes (see Sect. 3 for the definition of circularity) and extend it to our setting of tesserae. To each subset $X \subseteq \mathcal {B}^n$ a directed graph $\mathcal {G}(X)$ will be associated as the union of disjoint components $\mathfrak {C}_j(X)$ where $1 \le j \le \lfloor \frac{n}{2} \rfloor$. The vertices of such a component $\mathfrak {C}_j(X)$ will be initial segments and end segments of n-tuples from *X* of length *l* and $n-l$, respectively.

#### Definition 2.3

Let $n \in \mathbb {N}$ and $X \subseteq \mathcal {B}^n$. For $1 \le j \le \lfloor \frac{n}{2} \rfloor$ we define a graph component $\mathfrak {C}_{j}(X)=(V_j(X),E_j(X))$ with set of vertices $V_j(X)$ and set of arcs $E_j(X)$ as follows:

- $$V_j(X){:}{=}\{ N_1\cdots N_j, N_{j+1}\cdots N_n, N_1 \cdots N_{n-j}, N_{n-j+1} \cdots N_n \,:\, N_1N_2N_3\cdots N_n \in X\}$$
- $$E_j(X){:}{=}\{ N_1\cdots N_j \rightarrow N_{j+1}\cdots N_n, N_1\cdots N_{n-j} \rightarrow N_{n-j+1}\cdots N_n \,:\, N_1N_2N_3\cdots N_n \in X\}$$

The graph $\mathfrak {C}(X)$ associated to *X* is the union $\mathfrak {C}(X)=\bigcup \limits _{1 \le j \le \lfloor \frac{n}{2} \rfloor } \mathfrak {C}_j(X)$ of the graphs $\mathfrak {C}_{j}(X)$ for all $1 \le j \le \lfloor \frac{n}{2} \rfloor$. The graph $\mathfrak {C}(X)$ is called the *representing graph* of *X*.

**Fig. 3:**
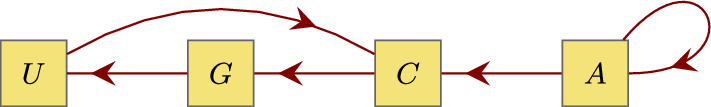
Graphical representation $\mathcal {G}(X)$ of the dinucleotide code X = {*UC, CG, GU, AC, AA*} which has only one component $\mathfrak {C}_1(X)$. (Color figure online)

**Fig. 4:**
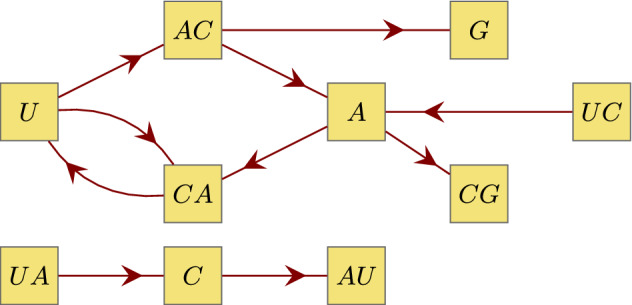
Graphical representation $\mathcal {G}(X)$ of the trinucleotide code X = {*UCA, UAC, CAU, ACA, ACG*} which has only one component $\mathfrak {C}_1(X)$ that is not connected. (Color figure online)

**Fig. 5:**
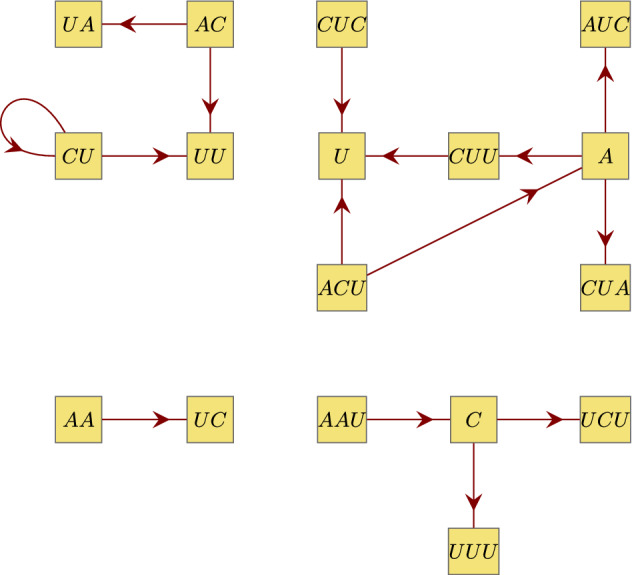
Graphical representation $\mathcal {G}(X)$ of the tetranucleotide code X = {*AAUC, ACUA, ACUU, CUCU, CUUU*} which has two components $\mathfrak {C}_1(X)$ and $\mathfrak {C}_2(X)$ that are both not connected but have two components themselves. (Color figure online)

It is easy to see that the graph components $\mathfrak {C}_j(X)$ of a representing graph $\mathcal {G}(X)$ are pairwise disjoint since their labels have different lengths. However, the components need not be connected. For the convenience of the reader and for a better illustration we give some examples for $n=2,3$ and 4 (Figs. 3, 4 and 5).

Since the tesserae are tetranucleotides it follows that any set of tesserae has two (maybe empty) graph components in their representing graph, one with labels of length 1 and 3 and the other with labels of length 2.

In Fimmel et al. (2016) the graph approach was used to characterize *circularity* of codes in terms of graph theory. We will consider circular tessera codes in the next section but it seems reasonable to state the corresponding theorem in this section. For the technical definition of circularity see Definition 3.1.

#### Theorem 2.4

Let $X \subseteq \mathcal {B}^n$. Then the following are equivalent:

1. *X* is a circular code;
2. the representing graph $\mathcal {G}(X)$ is acyclic, i.e. does not contain any cycle.

In the particular case of tesserae we will use a second graph associated to a set that we shall utilize later on in order to construct maximal circular tessera codes.

#### Definition 2.5

Let $X \subseteq TESS$. The *di-cut-graphs* $\mathcal {T}_{1,3}(X)$ and $\mathcal {T}_{2,4}(X)$ associated to *X* are defined as the representing graphs $\mathcal {G}(X_{1,3})$ and $\mathcal {G}(X_{2,4})$ of the sets

$$\begin{aligned} X_{1,3}=\{N_1N_3 \mid N_1N_2N_3N_4 \in X \} \end{aligned}$$

and

$$\begin{aligned} X_{2,4}=\{N_2N_4 \mid N_1N_2N_3N_4 \in X \} \end{aligned}$$

**Fig. 6:**
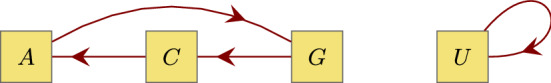
Graphical representation $\mathcal {U}_{1,3}(X)$ of the di-cut-graph of the Tessera code X = {UCUC, AUGC, CUAG, GCCG}. (Color figure online)

To conclude this section we give an example of a di-cut-graph $\mathcal {T}(X)$ of some tessera code *X* (Fig. 6).

## Circular Tessera Codes

In this section we consider circular tessera codes. Simply speaking circularity means that a frame-shift in any concatenation of tesserae from that code will be detected. In the biological setting of the genetic code, a circular set of trinucleotides was first observed in Arqués and Michel (1996) and is supposed to play an important role in error-detection mechanisms during the translational process. We start with the definition of circularity for tesserae.

### Definition 3.1

Let $n\in \mathbb {N}$. A tessera code $X \subseteq \mathcal {B}^4$ is called *n*-*circular* if for any set of tessera $t_i \in X$ $(1 \le i \le n)$ the concatenation $t_1 \ldots t_m$ has a unique decomposition into tesserae from the code *X* for any $m \le n$ if considered on the circle. We will call a tessera code $X \subseteq \mathcal {B}^4$ *circular*, if it is *n*-circular for all $n\in \mathbb {N}$.

As we had noted before in Theorem 2.4 a tessera code *X* is circular if and only if its representing graph $\mathcal {G}(X)$ is acyclic. Moreover, it is easy to see that the code *X* is *n*-circular if and only if for any concatenation $t_1 \cdots t_m$ of tesserae $t_1, \cdots , t_m$ from *X* with $m \le n$ the shifted sequences $\alpha _i(t_1 \cdots t_m)$ for $i \le 3$ do not yield a valid sequence in $X^m$, i.e.

$$\begin{aligned} \alpha _i(t_1 \cdots t_m) \not \in X^m \end{aligned}$$

In particular, a tessera code *X* is 1-circular if it does not contain the cyclically shifted tesserae of its members, i.e.

$$\begin{aligned} \alpha _i(t) \not \in X \end{aligned}$$

for all $i \le 3$ and $t \in X$. Therefore, a circular code can not contain any tessera that equals one of its shifts, e.g. $ACAC=\alpha _2(ACAC)$, and it makes sense to consider the equivalence classes that are formed by tesserae and their circular shifts. If all shifts are different, then this class is called *complete*. There are 12 such complete equivalence classes, each containing 4 elements. Four other classes each contain one element $\{AAAA\},\{CCCC\}, \{GGGG\}, \{UUUU\}$ and six classes each with two elements like $\{ACAC, CACA\}$. Table 5 displays all the complete equivalence classes of tesserae (Table 3).

**Table 3:** List of complete equivalence classes

Tessera	Shift 1	Shift 2	Shift 3	Class number
$\mathbf {AAUU}$	*AUUA*	$\mathbf{UUAA}$	*UAAU*	$D_1$
*AAGG*	*AGGA*	*GGAA*	*GAAG*	$D_2$
*AACC*	*ACCA*	*CCAA*	*CAAC*	$D_3$
$\mathbf{CCGG}$	*CGGC*	$\mathbf{GGCC}$	*GCCG*	$D_4$
*CCUU*	*CUUC*	*UUCC*	*UCCU*	$D_5$
*UUGG*	*UGGU*	*GGUU*	*GUUG*	$D_6$
$\mathbf{AGCU}$	*GCUA*	$\mathbf{CUAG}$	*UAGC*	$D_7$
$\mathbf{UGCA}$	*GCAU*	$\mathbf{CAUG}$	*AUGC*	$D_8$
$\mathbf{GUAC}$	*UACG*	$\mathbf{ACGU}$	*CGUA*	$D_9$
*AGUC*	*GUCA*	*UCAG*	*CAGU*	$D_{10}$
$\mathbf{UCGA}$	*CGAU*	$\mathbf{GAUC}$	*AUCG*	$D_{11}$
*ACUG*	*CUGA*	*UGAC*	*GACU*	$D_{12}$

Self-complementary tesserae are in bold

Since any circular code is also 1-circular and there are only 12 complete equivalence classes, it is obvious that a circular tessera code can contain at most 12 elements.

### Definition 3.2

A circular tessera code is called *maximal* if it contains exactly 12 elements.

We will show in Sect. 4 how to construct all maximal circular tessera codes and now give an example of a 1-circular tessera code that is not 2-circular.

### Example 3.3

Let $X=\{ ACGU, CAUG, GUCA, UGAC \}$. Then *X* is a 1-circular tessera code but the word *ACGUCAUG* has two decompositions on a circle

$$\begin{aligned} ACGU|CAUG \quad \textit{ and } \quad GUCA|UGAC=\alpha _2(ACGUCAUG). \end{aligned}$$

Thus *X* is not 2-circular. In particular, the graph component $\mathfrak {C}_2(X)$ of the representing graph of $\mathcal {G}(X)$ of *X* contains a cycle.

Moreover, the example below shows that also the classes of 2- and 3-circular tessera codes are different:

### Example 3.4

Let $X=\{ CAGU, UGCA, GUUG \}$. Then *X* is a 2-circular (by means of easy computations) but not a 3-circular tessera code since the word *CAGUUGCAGUUG* has two decompositions on a circle

$$\begin{aligned} CAGU|UGCA|GUUG \quad \textit{ and } \quad GUUG|CAGU|UGCA \end{aligned}$$

We show next that the graph component $\mathfrak {C}_2(G)$ being not acyclic is not an accident but in fact it is the only possibility for 1-circular codes not to be circular. In order to do so recall that a *cycle* in a graph $\mathcal {G}$ is a sequence $e_1 \rightarrow \cdots \rightarrow e_n \rightarrow e_1$ of **distinct** vertices $e_i$ $(i \le n)$ in $\mathcal {G}$. The length of this cycle is then defined to be *n*. Note that for $n=1$ a cycle of length 1 is a loop.

### Proposition 3.5

Let *X* be a tessera code. Then the following hold:

(i) The maximal length of a cycle in $\mathfrak {C}_1(X)$ is 2; in particular, the maximal length of a path that does not contain a cycle is 1;
(ii) The maximal length of a cycle in $\mathfrak {C}_2(X)$ is 4; in particular, the maximal length of a path that does not contain a cycle is 3.

### Proof

Let *X* be a tessera code. We first prove (*i*) by showing that any path in $\mathfrak {C}_1(X)$ of length 2 must contain a cycle. Hence assume that $\mathfrak {C}_1(X)$ contains a path of length 2. Without loss of generality we may assume that it starts with a nucleotide, e.g.

$$\begin{aligned} N_1 \rightarrow N_2N_3N_4 \rightarrow N_5. \end{aligned}$$

Then $N_1N_2N_3N_4$ and $N_2N_3N_4N_5$ are valid tesserae from *X*. By definition of tesserae the former tells us that there is a transformation $\alpha \in \mathcal {V}$ such that $\alpha (N_2)=N_4$ and $\alpha (N_3)=N_1$. The latter however, then implies that also $\alpha (N_3)=N_5$ and so $N_1=N_5$ which shows that $\alpha _1(N_1N_2N_3N_4)=N_2N_3N_4N_1 \in X$ and $N_1 \rightarrow N_2N_3N_4 \rightarrow N_1$ is a cycle.

We now prove (*ii*) by showing that any path of length 4 in $\mathfrak {C}_2(X)$ contains a cycle. Assume that $\mathfrak {C}_2(X)$ contains a path of length 4, e.g.

$$\begin{aligned} N_1N_2 \rightarrow N_3N_4 \rightarrow N_5N_6 \rightarrow N_7N_8 \rightarrow N_9N_{10}. \end{aligned}$$

By definition of $\mathcal {G}(X)$ there are permutations $\pi _1, \pi _2, \pi _3, \pi _4 \in \mathcal {V}$ such that

$$\begin{aligned}&\pi _1(N_1N_2)=N_3N_4, \quad \pi _2(N_3N_4)=N_5N_6, \quad \\&\quad \pi _3(N_5N_6)=N_7N_8, \quad \pi _4(N_7N_8)=N_9N_{10} \end{aligned}$$

If one of the $\pi _i$ is the identity we obtain a cycle of length 1 (a loop). Thus all $\pi _i$ are different from the identity. If $\pi _1=\pi _2$, then $N_1N_2=N_5N_6$ since $\pi _1^2=I$. This gives a cycle of length 2. Thus $\pi _1 \not =\pi _2$ and similarly $\pi _2\not = \pi _3$, $\pi _3 \not = \pi _4$. If $\pi _1 \not = \pi _3$, then the group structure of $\mathcal {V}$ implies that $\pi _1 \circ \pi _2 = \pi _3$ and so $N_7N_8=N_1N_2$, hence we obtain a cycle of length 3. Finally, if $\pi _1=\pi _3$, then similar arguments as above show that we get a cycle of length 3 or $\pi _2=\pi _4$ holds. Now

$$\begin{aligned} \pi _4(\pi _3(\pi _2(\pi _1(N_1N_2))))=\pi _2(\pi _1(\pi _2(\pi _1(N_1N_2))))=N_9N_{10} \end{aligned}$$

but $\mathcal {V}$ is commutative and all elements in $\mathcal {V}$ are of order 2, hence

$$\begin{aligned} N_9N_{10}=\pi _2(\pi _1(\pi _2(\pi _1(N_1N_2))))=N_1N_2. \end{aligned}$$

Consequently, the path itself is a cycle of length 4. $\square$

As a corollary we obtain an important theorem. Note that part (*ii*) was also obtained in a bachelor-thesis (Cisowski 2015) with a much more technical proof.

### Theorem 3.6

Let *X* be a tessera code. Then the following hold:

(i) If *X* is 1-circular, then $\mathfrak {C}_1(X)$ is acyclic;
(ii) The following two conditions are equivalent:
  - *X* is circular;
  - *X* is 3-circular.

### Proof

We first prove (i). By Proposition 3.5 we know that the maximal length of a cycle in $\mathfrak {C}_1(X)$ is 2, hence a cycle would be of the form $N_1N_2 \rightarrow N_3N_4 \rightarrow N_1N_2$ which contradicts 1-circularity since $\alpha _2(N_1N_2N_3N_4)=N_3N_4N_1N_2$.

In order to prove (ii) note that by Proposition 3.5 the maximal length of a cycle in $\mathcal {G}(X)$ is 4. However, a cycle of even length 2 is excluded by 1-circularity and of length 4 by 2-circularity since

$$\begin{aligned} N_1N_2 \rightarrow N_3N_4 \rightarrow N_5N_6 \rightarrow N_7N_8 \rightarrow N_1N_2 \end{aligned}$$

implies that $N_1N_2N_3N_4|N_5N_6N_7N_8$ has two decompositions - a contradiction. Hence $\mathcal {G}(X)$ does not contain any cycle of even length and the maximal length of an odd cycle is 3. By Theorem 2.3 from [13] we conclude that *X* is circular if and only if it is 3-circular. $\square$

We conclude this section with a result that gives a handy criterion for constructing circular tessera codes and some application.

### Theorem 3.7

Let $X \subseteq TESS$ be a tessera code. Then *X* is circular if

- *X* is 1-circular
- One of the di-cut graphs $\mathcal {T}_{1,3}(X)$ and $\mathcal {T}_{2,4}(X)$ is acyclic.

### Proof

Assume that *X* is 1-circular and one of the di-cut graphs $\mathcal {T}_{1,3}(X)$ and $\mathcal {T}_{2,4}(X)$ is acyclic. Without loss of generality we assume that $\mathcal {T}_{1,3}(X)$ is acyclic. Assume that *X* is not circular. Then Proposition 3.5 and Theorem 3.6 imply that the component $\mathfrak {C}_1(X)$ is acyclic and the maximal length of a cycle in $\mathfrak {C}_2(X)$ is 4. Assume without loss of generality that

$$\begin{aligned} N_1N_2 \rightarrow N_3N_4 \rightarrow N_5N_6 \rightarrow N_7N_8 \rightarrow N_1N_2 \end{aligned}$$

is a cycle in $\mathcal {G}(X)$. Thus the tesserae $N_1N_2N_3N_4, N_3N_4N_5N_6, N_5N_6N_7N_8$ and $N_7N_8N_1N_2$ are in *X*. By definition of $\mathcal {T}_{1,3}(X)$ is follows that $N_1N_3, N_3N_5, N_5N_7$ and $N_7N_1$ are dinucleotides in the set $X_{1,3}$ and hence $N_1, N_3, N_5$ and $N_7$ are vertices of $\mathcal {T}_{1,3}(X)$. Moreover,

$$\begin{aligned} N_1 \rightarrow N_3 \rightarrow N_5 \rightarrow N_7 \rightarrow N_1 \end{aligned}$$

is a cycle in $\mathcal {T}(X)$ - a contradiction to the fact that $\mathcal {T}_{1,3}(X)$ is acyclic. $\square$

The converse of Theorem 3.7 does not hold as the following example shows. Note, however, that the code $X_{1,3}$ (respectively $X_{2,4}$) can never contain dinucleotides of the form *NN* since they would imply that there is a tessera of the form *NKNK* in *X* which contradicts 1-circularity.

### Example 3.8

Let

$$\begin{aligned}&X=\{ AGUC, GAAG, CAAC, GGCC, AGCU, UGCA, GUAC, UUAA, CGAU, \\&\quad GACU, CUUC, GUUG \}, \end{aligned}$$

then *X* is a maximal circular tessera code but neither $\mathcal {T}_{1,3}(X)$ nor $\mathcal {T}_{2,4}(X)$ is acyclic.

We now state some application of the above results in order to construct maximal circular tessera codes from circular dinucleotide codes. In fact, the constructed codes will even have stronger properties:

### Definition 3.9

A circular tessera code $X \subseteq TESS$ is called a $C^4$-*code* if also the three shifted codes $\alpha _1(X)$, $\alpha _2(X)$ and $\alpha _3(X)$ are circular.

Recall from Fimmel et al. (2015) that a maximal circular dinucleotide code $D \subseteq \mathcal {B}^2$ must be of the form $D=\{N_1N_2, N_1N_3, N_1N_4, N_2N_3, N_2N_4, N_3N_4 \}$ where $N_1> N_2> N_3 > N_4$ is any linear ordering of the genetic alphabet $\mathcal {B}$.

### Proposition 3.10

Let $D=\{N_1N_2, N_1N_3, N_1N_4, N_2N_3, N_2N_4, N_3N_4 \}$ be a maximal circular dinucleotide code. Then

$$\begin{aligned} X= & {} \{ N_1N_1N_2N_2, N_1N_1N_3N_3, N_1N_1N_4N_4\} \cup \\&\cup \{ N_1N_3N_2N_4, N_1N_4N_2N_3 \} \cup \\&\cup \{ N_1N_2N_3N_3, N_1N_4N_3N_2 \} \cup \\&\cup \{ N_1N_3N_4N_2, N_1N_2N_4N_3 \} \cup \\&\cup \{ N_2N_2N_3N_3, N_2N_2N_4N_4, N_3N_3N_4N_4 \} \end{aligned}$$

is a maximal tessera $C^4$-code such that $\mathcal {T}_{1,3}(X)=\mathcal {G}(D)$.

### Proof

We first prove circularity of the code *X*. Clearly, $\mathcal {T}_{1,3}(X)=\mathcal {G}(D)$. Since *D* is circular its graph $\mathcal {G}(D)$ is acyclic by Theorem 2.4 and thus we only need to verify that *X* is 1-circular by Theorem 3.7. But this is clear since the code contains exactly one tessera from each of the twelve complete equivalence classes from Table 5.

Now let $X^{(n)}$ be the *n*th shift of *X* for $n \le 3$. Then we have

$$\begin{aligned} X^{(1)}_{1,3}= & {} \{N_1N_2, N_1N_3, N_1N_4, N_3N_4, N_4N_3, N_2N_3, N_4N_2, N_3N_2, N_2N_4 \} \\ X^{(2)}_{1,3}= & {} \{ N_2N_1, N_3N_1, N_4N_1, N_3N_2, N_4N_2, N_4N_3 \} =\overleftarrow{{X_{1,3}}}=\overleftarrow{{D}}\\ X^{(3)}_{1,3}= & {} \{ N_2N_1, N_3N_1, N_4N_1, N_4N_3, N_3N_4, N_3N_2, N_2N_4, N_4N_2, N_4N_3 \} = \overleftarrow{{X^{(1)}_{1,3}}} \end{aligned}$$

Clearly, $X^{(2)}_{1,3}$ is a dinucleotide circular code since it is equal to $\overleftarrow{{D}}$, hence its representing graph $\mathcal {G}(X^{(2)}_{1,3})=\mathcal {T}_{1,3}(X^{(2)})$ is acyclic and as above $X^{(2)}$ is 1-circular. By Theorem 3.7 we conclude that $X^{(2)}$ is a circular code.

It remains to show that also $X^{(1)}$ and $X^{(3)}$ are circular. However, in this case

$$\begin{aligned} X^{(1)}_{2,4}=\overleftarrow{{X_{1,3}}}=\overleftarrow{{D}} \end{aligned}$$

which is circular and so Theorem 3.7 implies that also $X^{(1)}$and also $X^{(3)}$ are circular. Hence *X* is a $C^4$-code. $\square$

We would like to remark that the construction in the above lemma has some flexibility, e.g the tessera of the form $N_iN_iN_jN_j$ can be substituted by tessera from the same equivalence class. However, it is not obvious how to construct all maximal circular tessera codes using this method. Nevertheless, in the next section we will give a way to obtain all such codes.

## Construction of All Maximal Circular Tessera Codes

**Fig. 7:**
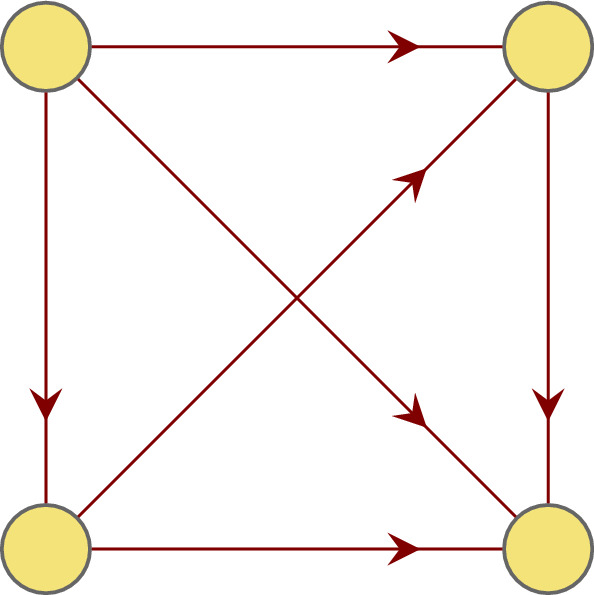
An acyclic tournament on four nodes. (Color figure online)

This section introduces one possibility to construct all maximal circular tessera codes. Recall that a circular tessera code is maximal if it contains exactly 12 elements. The construction will be accomplished in two major steps. Firstly, for each of the four equivalence classes from Table 1 we define a tournament on four vertices which are representing the single dinucleotides. Finally, we combine the four tournaments constructed in the previous step to construct maximal circular tessera codes. Recall that a tournament is a complete oriented graph (see e.g. Clark and Holton 1991). Figure 7 shows an example of a tournament.

As already proved in Theorem 3.6, the graph component $\mathfrak {C}_{1}(X)$ associated to a tessera code *X* has either no path bigger than 1 or *X* is not circular. Even more precise, if $\mathfrak {C}_{1}(X)$ is acyclic the code *X* must not even be 1-circular. Considering that, a construction of a maximal circular tessera code could almost be reduced to the problem of constructing a valid and acyclic $\mathfrak {C}_{2}$ which represents a correct tessera code *X*.

Step 1: In this step we construct four acyclic tournaments which together represent a tessera code $X'$ of length 24 so that $\mathfrak {C}_{2}(X')$ is acyclic. Note that a tournament on 4 vertices has exactly 6 edges and in order to be acyclic it has to be isomorphic to the tournament given in Fig. 7. Below we will show how to construct tournaments on four vertices that represent a correct (circular) tessera code, i.e. the tournaments will be acyclic. Together they form the desired code $X'$ as
  1 $$\begin{aligned} X'= & {} X_{I} \cup X_{SW} \cup X_{YR} \cup X_{KM} \end{aligned}$$
  2 $$\begin{aligned} \text { with } \quad |X_{I}|= & {} |X_{SW}| = |X_{YR}| = |X_{KM}| = 6\quad \text {and, thus, }\quad |X'| = 24 \end{aligned}$$
  As it can be seen from the construction, $\mathfrak {C}_{2}(X')$ is acyclic as it is the union of acyclic tournaments, while $\mathfrak {C}_{1}(X')$ is not. Yet, for this initial step we can ignore this fact. Since $\mathfrak {C}_{2}(X_{I})$, $\mathfrak {C}_{2}(X_{SW})$, $\mathfrak {C}_{2}(X_{YR})$ and $\mathfrak {C}_{2}(X_{KM})$ are disjoint it is sufficient that these subgraphs are acyclic to ensure the acyclicity of $\mathfrak {C}_{2}(X')$. As mentioned above, each of these subgraphs has to be isomorphic to the graph in Fig. 7.
  Let us choose one of the equivalence classes $\Sigma _{i}, i\in \{I,SW,YR,KM\}$ and assign numbers 1, 2, 3, 4 to the dinucleotides of $\Sigma _{i}$. Now we draw directed edges from each node to the nodes with a higher number. This way we will obtain four acyclic tournaments, each of them represents a circular tessera code of size 6. This gives 4! possible assignments per subgraph. Hence, there are altogether $(4!)^4=331776$ tessera codes of size 24 with an acyclic $\mathfrak {C}_{2}$-component.
Step 2: In this step, we use the 331776 tessera codes, constructed in Step 1, to construct all possible maximal circular tessera codes. Since the $\mathfrak {C}_{2}$ is already acyclic, it is sufficient to focus on $\mathfrak {C}_{1}$.

### Lemma 4.1

Let $X'$ be a tessera code constructed as above and

$$\begin{aligned} t = N_1N_2N_3N_4 = N_1N_2\gamma (N_1N_2)\in X' \end{aligned}$$

for some $\gamma \in \mathcal {V}$. Then the following hold:

1. $$\alpha _2(t)\notin X'$$
2. $$ \alpha _3(t) = N_4N_1N_2N_3 \in X' \text { or } \alpha _1(t) = N_2N_3N_4N_1 \in X'$$.

### Proof

First we prove (1). Obviously, *t* is represented by the arrow $N_1N_2\rightarrow N_3N_4$ in the corresponding tournament. Obviously, $\gamma \ne id$. Let us consider $\alpha _2(t) = N_3N_4N_1N_2$. It follows that $\alpha _2(t)\notin X'$ since it would be represented in the same tournament by the opposite directed arrow $N_4N_3\rightarrow N_1N_2$ - a contradiction. Now we claim that one of the remaining shifts of *t*

$$\begin{aligned} \alpha _3(t) = N_4N_1N_2N_3 \text { or } \alpha _1(t) = N_2N_3N_4N_1 \end{aligned}$$

is necessarily in the code $X'$. Let us first assert that the dinucleotides $N_4N_1$ and $N_2N_3$ cannot be in the same equivalence class as $N_1N_2$ and $N_3N_4$ since in this case $N_4=N_2$ takes place and, thus, $\gamma =id$. Consequently, one of the arrows $N_4N_1\rightarrow N_2N_3$ or $N_2N_3\rightarrow N_4N_1$ is drawn in the corresponding tournament and it follows that $\alpha _3(t)\in X'$ or $\alpha _1(t)\in X'$. This proves (2). $\square$

The above lemma shows that consequently, $X'$ consists of 12 pairs of cyclically equivalent tesserae. To ensure that the codes are circular, one of the cyclically equivalent tuples must be removed. This has to be done for all 12 cyclically equivalent pairs of tuples in such a code $X'$. It follows that each of the 331776 codes can be used to construct $2^{12}$ circular codes - with possible repetitions. It remains to prove that all maximal circular tessera codes can be obtained this way. Let *X* be a auch a maximal code. As shown above, the $\mathfrak {C}_2$ component of each $X_i, i \in \{I, KM, SW, YR\}$ is a simple directed acyclic graph with a maximum of four nodes. According to Theorem 3.1 (Fimmel et al. 2017), such a graph can be embedded in an acyclic tournament. In Step 1, all possible acyclic tournaments are constructed. Step 2 takes all possible subgraphs of each tournament and combines those. This ensures that all possible maximal circular tessera codes are represented in the construction.

Hence, the total amount of $2^{12}\times {}(4!)^4 = 1358954496$ constructed maximal circular tessera codes include all maximal circular tessera codes.

The table below gives the exact numbers of circular and even $C^4$- codes (compare 3.9) for all cardinalities from 1 to the maximum 12. Moreover, it also shows that number of *comma-free* codes. Recall that comma-free codes form a subclass of circular codes.

### Definition 4.2

A code $X \subseteq \mathcal {B}^l$ is called *comma-free* if any concatenation $x_1x_2$ does not contain any $x \in X$ as a substring except for $x_1$ (as initial segment) and $x_2$ (as end segment) themselves.

Clearly, a comma-free code is circular and *X* is comma-free if and only if it associated graph has no path of length more than 2 (see Fimmel et al. 2016) (Table 4).

**Table 4:** Numbers of circular, comma-free and $C^4$-tessera codes of different code lengths

Code length	# 1-circular codes	# Circular codes	# $C^4$-codes	# Comma free codes
1	48	48	48	48
2	1056	1056	1056	1056
3	14080	14048	14016	13952
4	126720	125544	124368	122376
5	811008	791952	773088	745584
6	3784704	3606048	3433584	3214272
7	12976128	11908800	10922112	9816960
8	32440320	28230456	24577404	20952504
9	57671680	46720800	37987120	30297824
10	69206016	51111024	38129856	28015728
11	50331648	33113472	22240992	14790144
12	16777216	9592512	5685408	3351232

### Self-Complementary Circular Tessera Codes

In this section we will discuss some properties of self-complementary tessera codes. In particular, we will determine all maximal self-complementary comma-free tessera codes and give a graph-theoretical characterization of self-complementarity for tessera codes.

Let us first recall the definition of self-complementarity of a code.

#### Definition 4.3

Let $X\subseteq \mathcal {B}^\ell$ be a $\ell$-nucleotide code. We will call *X* self-complementary if for each $\ell$-nucleotide $x\in X$ its anti-$\ell$-nucleotide $\overleftarrow{{SW(x)}}$ is also in *X*:

$$\begin{aligned} x\in X\Leftrightarrow \overleftarrow{{SW(x)}}\in X. \end{aligned}$$

We will also use the notation

$$\begin{aligned} X=\overleftarrow{{SW(X)}}. \end{aligned}$$

According to the above, a circular tessera code can contain a maximum of 12 tesserae. Such a code can even be self-complementary, as the next example shows.

#### Example 4.4

The following code $X \subseteq TESS$ is a self-complementary maximal circular code:

$$\begin{aligned}&X=\{AAUU, CCGG, AGCU, UGCA, GUAC, UCGA, AAGG, \\&\quad CCUU, AACC, GGUU, AGUC, GACU\}. \end{aligned}$$

The next lemma gives the exact number of self-complementary 1-circular tessera codes.

#### Lemma 4.5

The maximal size of a self-complementary 1-circular tessera code is 12 and the number of them is 4096.

#### Proof

Firstly, Example 4.4 shows that there are self-complementary circular codes of size 12 which is maximal. Secondly, inn order to calculate the exact number of self-complementary 1-circular codes, we first ascertain that for 6 conjugacy classes, the respective antitessera of a tessera from that class is found in another conjugacy class: The antitesserae of tesserae from class $D_2$ are all in class $D_5$, from class $D_3$ in class $D_6$ and from class $D_{10}$ in class $D_{12}$ and, of course, vice versa. Thus, we have $4^3$ possibilities to choose 6 tesserae from these conjugacy classes for a 1-circular self-complementary tessera code. As for the classes $D_1, D_4, D_7, D_8, D_9, D_{11}$, only the self-complementary tesserae can be chosen from these, since the other two form tessera-antitessera pairs and are cyclically equivalent. So we have further $2^6$ possibilities for this. Altogether we have $2^6\cdot 4^3=4096$ maximal self-complementary 1-circular codes. $\square$

The following example shows that not every 1-circular self-complementary tessera code is also circular (even not 2-circular).

#### Example 4.6

Let us take (self-complementary) tesserae *AAUU* from the class $D_1$ and *CCGG* from the class $D_4$, as well as *GGAA* (from $D_2$) and *UUCC* (from $D_5$) which are complementary to each other. Then we have that the word *CCGGAAUU* has two different decompositions on a circle:

$$\begin{aligned} CCGG|AAUU\quad \text{ and }\quad CC|GGAA|UU. \end{aligned}$$

With an extensive computer calculation the exact numbers of self-complementary circular and comma-free codes of maximal length are calculated:

#### Lemma 4.7

There are

- 1584 self-complementary circular (Table 5) and
- 16 self-complementary comma-free (Table 6)

tessera codes of maximal length.

**Table 5:** Numbers of self-complementary circular codes of different code lengths

Code length	1	2	3	4	5	6	7	8	9	10	11	12
Number	12	72	304	996	2580	5408	9264	12708	13696	11232	6144	1584

**Table 6:** The list of all self-complementary comma-free tessera codes of maximal length

UUAA	CCAA	AGGA	UCCU	UUGG	CCGG	UCGA	CAUG	ACGU	AGCU	ACUG	CAGU
AAUU	AACC	AGGA	UCCU	GGUU	GGCC	UCGA	CAUG	ACGU	AGCU	ACUG	CAGU
UUAA	CCAA	GAAG	CUUC	UUGG	CCGG	GAUC	CAUG	ACGU	CUAG	ACUG	CAGU
AAUU	AACC	GAAG	CUUC	GGUU	GGCC	GAUC	CAUG	ACGU	CUAG	ACUG	CAGU
UUAA	CCAA	AGGA	UCCU	UUGG	CCGG	UCGA	UGCA	GUAC	AGCU	UGAC	GUCA
AAUU	AACC	AGGA	UCCU	GGUU	GGCC	UCGA	UGCA	GUAC	AGCU	UGAC	GUCA
UUAA	CCAA	GAAG	CUUC	UUGG	CCGG	GAUC	UGCA	GUAC	CUAG	UGAC	GUCA
AAUU	AACC	GAAG	CUUC	GGUU	GGCC	GAUC	UGCA	GUAC	CUAG	UGAC	GUCA
AAUU	ACCA	AAGG	CCUU	UGGU	CCGG	GAUC	UGCA	ACGU	AGCU	GACU	AGUC
UUAA	ACCA	GGAA	UUCC	UGGU	GGCC	GAUC	UGCA	ACGU	AGCU	GACU	AGUC
AAUU	CAAC	AAGG	CCUU	GUUG	CCGG	GAUC	CAUG	GUAC	AGCU	GACU	AGUC
UUAA	CAAC	GGAA	UUCC	GUUG	GGCC	GAUC	CAUG	GUAC	AGCU	GACU	AGUC
AAUU	ACCA	AAGG	CCUU	UGGU	CCGG	UCGA	UGCA	ACGU	CUAG	CUGA	UCAG
UUAA	ACCA	GGAA	UUCC	UGGU	GGCC	UCGA	UGCA	ACGU	CUAG	CUGA	UCAG
AAUU	CAAC	AAGG	CCUU	GUUG	CCGG	UCGA	CAUG	GUAC	CUAG	CUGA	UCAG
UUAA	CAAC	GGAA	UUCC	GUUG	GGCC	UCGA	CAUG	GUAC	CUAG	CUGA	UCAG

We now aim for a graph-theoretical characterization of self-complementarity for tessera codes. Let us start with some observations on self-complementary 1-circular tessera codes:

#### Lemma 4.8

Let $X\subseteq TESS$ be a self-complementary 1-circular tessera code. Then it holds

$$\begin{aligned} X_{SW}=\emptyset . \end{aligned}$$

#### Proof

Let *X* be a self-complementary 1-circular tessera code. Then for all $t=d_1d_2\in X_{SW}$

$$\begin{aligned} \overleftarrow{{c(t)}}=\overleftarrow{{c(d_2)}}\overleftarrow{{c(d_1)}}= d_2d_1=\alpha _2(t) \end{aligned}$$

where $d_1,d_2\in \Sigma _{SW}$. However, cyclically equivalent tesserae cannot be in the same 1-circular code. $\square$

The next property is discovered by examining maximum circular codes of codons (RNA triplets) (Fimmel et al. 2018). Assume that $Y\subset \mathcal {B}^3$ is a trinucleotide self-complementary code, $\mathcal {G}(Y)=(V,E)$ the graph associated to *Y*. Then the following conditions are true:

1. $$V= \overleftarrow{{c(V)}}$$
2. $d^+(v)=d^-(\overleftarrow{{c(v)}})$ *for all vertices* $v \in V$

where $d^+(v)$ of a vertex *v* denotes the number of *outgoing* edges (directed edges that start in *v*) and $d^-(v)$ denotes the number of *ingoing* edges, respectively. It was also shown in Fimmel et al. (2018) that the conditions from above are not sufficient in general to ensure self-complementarity but only for circular codes of size at least 18.

We will show next that in the case of tesserae or dinucleotides, the size of the code does not matter and that one can obtain a similar result. Let us first prove the claim for dinucleotides:

#### Lemma 4.9

Let $X\subseteq \mathcal {B}^2$ be a 1-circular dinucleotide code, $\mathcal {G}(X)=(V,E)$ its associated graph. *X* is self-complementary if and only if

1. $$V= c(V)$$
2. $d^+(v)=d^-(c(v))$ *for all vertices* $v \in V$

#### Proof

Let *X* be a self-complementary dinucleotide code, $l_1l_2\in X$ for some $l_1l_2\in \mathcal {B}$. Due to self-complementarity of *X* we have $c(l_2)c(l_1)\in X$ which implies that both conditions (1) and (2). Conversely, assume that *X* is a 1-circular code. Then its associated graph $\mathcal {G}(X)$ can be embedded into a tournament on four vertices $A,C,G,U\in \mathcal {B}$ (compare Fimmel et al. 2017). Assume that $\mathcal {G}(X)$ satisfies the conditions (1) and (2). The presence or absence of the self-complementary dinucleotides *AU*, *UA*, *CG* or *GC* in *X* does not affect either the self-complementarity of *X* or the conditions (1) and (2). Let us focus then on non-self-complementary dinucleotides from *X*. Suppose without loss of generality that the dinucleotide $A\rightarrow C$ is in the code. For conditions (1) and (2) to be met, a dinucleotide $N_1U$ and a dinucleotide $GN_2$ must be in the code. This can be achieved in three ways:

- $N_1=G, N_2=U$ In this case $AC=\overleftarrow{{c(GU)}}$ is valid or
- $N_1=C, N_2=U$ The condition (2) can now only be met if the dinucleotide $AG\in X$ and the code is self-complementary or
- $N_1=C, N_2=A$ The condition (2) can now only be met if the dinucleotide $UG\in X$ and the code is self-complementary

This proves that *X* is self-complementary. $\square$

In the case of tesserae we should additionally consider the condition from the Lemma 4.8 and obtain a handy characterization of self-complementarity.

#### Theorem 4.10

Let $X\subseteq TESS$ be a 1-circular tessera code, $\mathfrak {C}_{2}(X)=(V_2,E_2)$. *X* is self-complementary if and only if

1. $$X_{SW}=\emptyset $$
2. $$V_2= \overleftarrow{{c(V_2)}}$$
3. $d^+(v)=d^-(\overleftarrow{{c(v)}})$ *for all vertices* $v \in V_2$

#### Proof

One implication is analogous to the proof of Proposition 3.1 in Fimmel et al. (2018) considering Lemma 4.8. Conversely, assume that $X\subseteq TESS$ is a 1-circular tessera code that satisfies all three conditions (1), (2), (3). It is immediately clear by direct verification that for all equivalence classes $\Sigma _i$ with $i\in \{I, SW, YR, KM\}$

$$\begin{aligned} \overleftarrow{{c(\Sigma _i)}}=\Sigma _i \end{aligned}$$

holds, i.e. the dinucleotide codes $\Sigma _i$ are self-complementary. So we can restrict ourselves to the consideration of $\mathfrak {C}_2 (X_i)$ for $i\in \{I, SW, YR, KM\}$. Since *X* is a 1-circular code each of $\mathfrak {C}_2 (X_i)$ is embedded into a tournament on four nodes.

Secondly, as we can see from Table 1, two of the six tesserae represented in each tournament, except of that corrsponding to $\Sigma _{SW}$, are self-complementary:

- For $\Sigma _I$ these are *AAUU* (or *UUAA*) and *CCGG* (or *GGCC*)
- For $\Sigma _{YR}$ these are *ACGU* (or *GUAC*) and *UGCA* (or *CAUG*)
- For $\Sigma _{KM}$ these are *AGCU* (or *CUAG*) and *UCGA* (or *GAUC*)

and for each not self-complementary tessera $T=d_1d_2\in X_i$ where $i\in \{I, SW, YR, KM\}$ its anti-tessera should be in the same component $X_i$ due to the fact that

$$\begin{aligned} \overleftarrow{{c(U)}}=\overleftarrow{{c(d_2)}}\overleftarrow{{c(d_1)}}. \end{aligned}$$

The rest of the proof can now be done analogously to the proof of Lemma 4.9.

$$\square $$

In the Theorem above, the condition of 1-circularity can not be omitted, as the following example shows:

#### Example 4.11

Let us consider the following tessera code

$$\begin{aligned} X=\{CUGA, GACU, AGAG, UCUC\}. \end{aligned}$$

The code is obviously not 1-circular and non-self-complementary since, for instance, $\overleftarrow{{c(AGAG)}}=CUCU\notin X$ takes place. But all three conditions from Theorem 4.10 are fulfilled. In the picture below, the round and square nodes represent pairs of reversed-complementary dinucleotides.

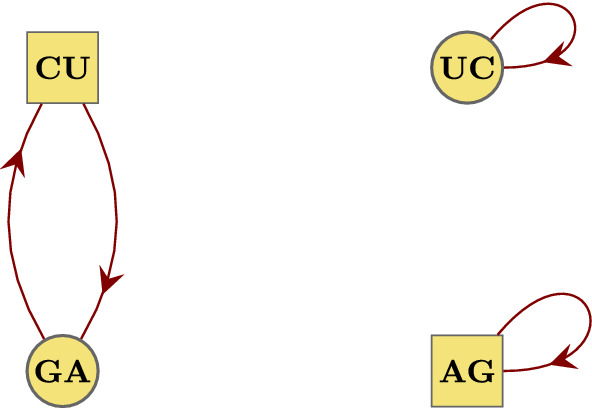



We conclude this section with a second theorem that gives a graph-theoretical characterization for tessera codes that are not 1-circular using the graph component $\mathfrak {C}_{1}(X)$ of a code *X*.

#### Theorem 4.12

Let $X\subseteq TESS$ be a tessera code, $\mathfrak {C}_{1}(X)=(V_1,E_1)$. *X* is self-complementary if and only if

1. $$V_1= \overleftarrow{{c(V_1)}}$$
2. $d^+(v)=d^-(\overleftarrow{{c(v)}})$ *for all vertices* $v \in V_1$

#### Proof

Let us assume that $X \subseteq TESS$ satisfies properties (1) and (2) from Theorem 4.12. Hence, for any tessera $N_1N_2N_3N_4 \in X$ we have that $N_2N_3N_4 \in V_1$ and by property (1) also $c(N_4N_3N_2) \in V_1$. Property (2) then implies that $c(N_4N_3N_2)N_5 \in X$ for some basis $N_5$. It is clear that $N_5$ has to be the complement of $N_1$ by the unique definition of tesserae. More precisely, assume that $\pi \in \mathcal {V}$ such that $N_2 = \pi (N_4)$ which implies that $c(N_2) = \pi (c(N_4))$ and thus $c(N_3) = \pi (N_5)$. Hence $N_5$ = $c(N_1)$. Therefore $c(N_4N_3N_2)N_5 = c(\overleftarrow{{N_1N_2N_3N_4}}) \in X$ and *X* is self-complementary. $\square$

Let us make a final remark: A 1-circular tessera code *X* represented by a tournament which is built on four dinucleotides of one of the equivalence classes (see Table 1) is self-complementary if and only if the numbers 1, 2, 3, 4 (see paragraph Construct a Tournament) are assigned to dinucleotides so that 1 is complementary to 4 and 2 is complementary to 3, i.e. $d_1=\overleftarrow{{c(d_4)}},\; d_2=\overleftarrow{{c(d_3)}}$. In order to see this let the order on dinucleotides be defined as described above, $d_id_j\in X, i<j, i,j\in \{1,2,3,4\}$ and

$$\begin{aligned} \overleftarrow{{c(d_id_j)}}=\overleftarrow{{c(d_j)}}\overleftarrow{{c(d_i)}}=d_kd_l. \end{aligned}$$

If $i=1$ or $j=4$ then it is obvious that $k<l$ since $k=1$ or $l=4$ and $d_kd_l\in X$. The only remaining case is $i=2, j=3$. But in this case $k=2, l=3$ takes place per definition of the order on dinucleotides and $d_2d_3\in X$. The opposite direction: Let $d_1=\overleftarrow{{c(d_2)}}$ and, correspondingly, $d_3=\overleftarrow{{c(d_4)}}$. Then $\overleftarrow{{c(d_1d_3)}}=d_4d_2\notin X$. The case $d_1=\overleftarrow{{c(d_3)}}$ is analogous. In both cases *X* is not a self-complementary code. Here is an example.

#### Example 4.13

For example, let us consider the class $\Sigma _{KM}$. Then one possible self-complementary assignment would be: $1\rightarrow CU$, $4\rightarrow AG$, $2\rightarrow UC$ and $3\rightarrow GA$. The represented code $X_{KM}=$ {*CUAG, CUUC, CUGA, UCAG, UCGA, GAAG*} is self-complementary.

This shows that in the construction of all maximal circular tessera codes one can also identify and construct all maximal self-complementary circular codes.

## Conclusions

In this work we have identified and characterized circular tessera codes and their properties. In Gonzalez et al. (2012) and Gonzalez et al. (2019) Gonzalez, Giannerini and Rosa had proposed an ancestor code of the universal genetic code that is based on 64 tetranucleotides built from dinucleotides by using the Klein four symmetry group. It was hypothesized that this *tessera code* existed before LUCA and even before the early genetic code that coded for 20 amino acids using all 64 codons. Possible primeval adaptor molecules that could decode the tessera were also modelled and it was shown that the tessera code mirrors exactly the degeneracy distribution of the mitochondrial genetic code.

We have combined the theory of tesserae with the the theory of circular codes that have been studied extensively during the last decades. Circular codes were found by an extensive statistical investigation in Arqués and Michel (1996) and seem to play an important role in the detection and correction mechanisms of the ribosome during translation. Moreover, it was hypothesized in [13] that ancestor codes of the universal genetic code might have used codons from a circular code only. Thus it was reasonable to investigate circular tessera codes which could have existed between a primitive genetic code and the tessera code.

Our results show that circular tessera codes can be of size 12 at most and we have given construction methods for all circular tessera codes of this size. Moreover, the number of circular (comma-free, self-complementary) tessera codes of any size between 1 and 12 have been calculated.
